# Life event stress is associated with blunted cardiovascular responding to both personally salient and personally non‐salient laboratory tasks

**DOI:** 10.1111/psyp.14199

**Published:** 2022-10-25

**Authors:** Siobhán Howard, Stephen Gallagher, Annie T. Ginty, Anna C. Whittaker

**Affiliations:** ^1^ SASHLab, Centre for Social Issues Research, Department of Psychology University of Limerick Limerick Ireland; ^2^ Health Research Institute University of Limerick Limerick Ireland; ^3^ Baylor Behavioral Medicine Lab Baylor University Waco Texas USA; ^4^ Faculty of Health Sciences and Sport University of Stirling Stirling UK

**Keywords:** blunted reactivity, cardiovascular reactivity, life event stress, personally salient stress

## Abstract

Life event stress has been associated with blunted cardiovascular reactivity to acute psychological stress. However, recent studies have suggested that blunted reactivity to stress only arises when the laboratory tasks are not personally salient to the individual. We re‐analyzed data from 136 healthy young adults where we had previously reported a negative association between life event stress and cardiovascular reactivity to two combined stressors. Participants completed a mental arithmetic task and a personally salient speech task, following a formal baseline period with Finometer‐assessed cardiovascular parameters. The reanalyses examined reactivity to the verbal mental arithmetic (personally non‐salient) and speech (personally salient) tasks separately and found that life event stress was negatively associated with diastolic blood pressure reactivity, to both the personally non‐salient, *β* = −.20, *p* = .023, and personally salient stressors, *β* = −.24, *p* = .004. Life event stress was negatively associated with systolic blood pressure reactivity to the personally salient stressor only, *β* = −.20, *p* = .021, and was not associated with heart rate reactivity. This study provides evidence against the argument that blunted reactivity to stress emerges as a result of stressor context, with findings indicating that low reactors show lower reactivity to both personally salient and personally non‐salient stress.

## Abstract 2

Recent research has identified that blunted cardiovascular reactions to stress may arise as laboratory tasks employed have low personal salience. Our research challenges this proposition. Our findings show that blunted cardiovascular reactions to stress arise to both personally salient and personally non‐salient stress‐tasks. Patterns of low or high reactivity exhibited by individuals maintained across both personally salient and non‐salient stressors.

## 1 INTRODUCTION

The association between physiological reactions to stress and health outcomes is a cornerstone of psychosomatic medicine. A key paradigm within the literature is the “reactivity hypothesis” where exaggerated and/or sustained cardiovascular reactions to stress have been shown to be prospectively associated with a range of indicators of cardiovascular diseases as well as disease mortality (e.g., Chida & Steptoe, 2010). Since its detailed explanation in the 1980s (Obrist, 1981), across 40 years of research, many studies have confirmed its prospective value in predicting a range of indicators of cardiovascular disease and disease endpoints (e.g., Barnett et al., 1997; Carroll, Ginty, Der, et al., 2012; Carroll, Ginty, Painter, et al., 2012). However, within the past two decades, the “blunted” model of reactivity has also shown that low, as well as high, cardiovascular reactions to stress are prospectively associated with negative health outcomes (e.g., Ahern et al., 1990; Carroll et al., 2008; de Rooij et al., 2010; O'Riordan et al., in press; Phillips et al., 2012), and poorer self‐reported health (de Rooij & Rosebook, 2010; Phillips et al., 2009). It is thought that blunted cardiovascular reactivity to stress is associated with poorer health via indirect routes, such as changes in health behavior, depression, and motivational dysregulation (Carroll et al., 2017).

Recently, the methodological details of how the blunted reactivity model arose have been queried. Bourassa and Sbarra (2022) have argued that the personal emotional salience of the stress‐tasks used in the literature may be an important moderator of the magnitude of the cardiovascular reaction to stress. Using a sample of 109 recently separated or divorced adults, a task with low personal salience was associated with blunted cardiovascular reactions; however, using a high personally salient task, greater divorce‐related distress was associated with higher blood pressure reactivity. Their findings, they argued, showed that the personal emotional salience of the laboratory task may influence the cardiovascular response which may have important implications for interpreting apparent discrepancies between the traditional and blunted models of cardiovascular reactivity. They argue that the types of tasks used may explain the blunted cardiovascular reactions exhibited when subgroups of a population engage with cognitive challenges such as mental arithmetic and Stroop tasks; tasks that are designed to have low personal emotional salience.

While the Bourassa and Sbarra's (2022) study is a welcome addition to the reactivity field, the data were limited by a number of over‐looked elements. Firstly, the divorce‐recall task did not elicit a significant cardiovascular response within the entire sample; that is, for the entire sample, recalling their divorce was not a physiologically stressful task for the group. Cardiovascular reactions were only evoked by divorce recall in those who reported high divorce stress, implying that the personal emotional salience of the stressor is the important feature for reactivity. This is a significant departure from the essence of the reactivity hypothesis, where tasks used to elicit a cardiovascular response to stress are usually shown to be stressful for the entire group. Indeed, many reactivity studies report this as the first manipulation check in their papers; analyses that confirm the stress‐task employed was successful in perturbing the cardiovascular system.

A second element of the “reactivity hypothesis” not acknowledged or discussed by the authors is that cardiovascular reactivity to stress is demonstrated to be a relatively stable individual difference (e.g., Ginty et al., 2013), a necessary feature in order to hold predictive value in the development of cardiovascular diseases. Therefore, the reactivity hypothesis is not based on the reactions to a stressor being reflective of how stressed that person is in response to that stressor; rather, it is reflective of that individual's usual cardiovascular response to psychological stress. Variations in the face of different stressors will occur within the individual, but on the whole, the magnitude of the response is reflective of that individual's usual response style, not their stress perception. Indeed many blunted reactivity studies show that the magnitude of the biological stress response is independent of the psychological perception of stressfulness (e.g., Bibbey et al., 2013; Heaney et al., 2011) or emotional responses to the task (Campbell & Ehlert, 2012). This trait‐like characteristic of reactivity has been demonstrated with cardiovascular reactivity in young adulthood predictive of reactivity in later adulthood (Hassellunud et al., 2010; Sherwood et al., 1997), as well as evidence of consistency in children and adult's hemodynamic responses to different laboratory stressors (Ginty et al., 2013, 2019; Musante et al., 1994).

The notion that the blunted model of reactivity may be reflective, not of stable individual differences in stress reactivity, but context‐dependent features of the stress‐tasks employed does deserve keen attention and is itself an empirical question. Therefore, to investigate if blunted reactivity to stress may be a feature of the personal salience of the task employed, we returned to a paper published from our lab reporting a pattern of blunted reactivity in a subgroup of individuals. Gallagher et al. (2018) identified that life events stress was associated with blunted cardiovascular reactivity to stress in a sample of 184 young adults. However, despite including two active stress situations with varying personal salience, reactivity was assessed using the mean reaction to both tasks; that is, reactivity was assessed as the mean elevation to both tasks relevant to baseline. This approach, taking the average reactivity to a number of tasks was originally a keystone of the reactivity hypothesis, with reactivity to a range of tasks recommended as a reliable means to assess reactivity as an individual difference trait (see Manuck, 1994). However, for the present paper and the empirical question posed, this study design offers an opportunity to specifically test if the personal salience of a stress‐task is an important feature where blunted reactions to stress arise.

Life events stress has previously been shown to be associated with blunted cardiovascular reactivity to stress in both middle‐aged and older adult cohorts (Carroll et al., 2005) as well as young adults (Phillips, Carroll, et al., 2005). More recently, studies from our laboratories have shown that greater frequency and perceptions of life stress was associated with blunted blood pressure and heart rate responses to stress, as well as impaired cardiovascular stress‐response habituation (Tyra et al., 2020). Similarly, other labs have reported a negative association between blood pressure and heart rate reactivity and life event stress, even in younger cohorts (Musante et al., 2000). However, a common thread across these studies is the use of a task with low personal salience (e.g., mental arithmetic, car driving simulation). Perhaps the association between life event stress and blunted cardiovascular reactions to stress across the lifespan is due to the use of tasks with low personal emotional salience. In fact, in Musante et al.’s study, those with high life event stress showed elevated cardiac output reactivity to the social competence interview; a stress‐task with high personal relevance.

Consequently, the aim of the present study was to examine if the personal salience of a laboratory stressor is a driver of blunted reactions to stress. We re‐analyzed published data, separating the cardiovascular reactions to a verbal mental arithmetic stressor from the cardiovascular reactions to a personally salient speech task. We aimed to identify if those who have experienced high life event stress exhibited blunted cardiovascular reactions to just the personally non‐salient stress or if those with high life event stress also exhibited blunted reactions to the personally salient stress; in essence, is it the case that once you are a blunted reactor, you remain a blunted reactor? We hypothesized that the personal salience of the stress‐task would *not* be an important moderator of the cardiovascular stress response.

## 2 METHOD

### 2.1 Participants

Participants were drawn from the sample reported by Gallagher et al. (2018). Inclusion criteria for the present study was resting systolic blood pressure (SBP) and diastolic blood pressure (DBP) < 140/90 mmHg, age < 30 years, and not taking medication that would affect cardiovascular reactivity. The sample consisted of 136 students (62% female), ranging in age from 18 years to 29 years (*M* = 20.67, *SD* = 2.22), with a mean body mass index of 23.58 kg/m^2^ (*SD* = 3.37). Over 96% reported their ethnicity as white/Caucasian, with two participants reporting their ethnicity as Asian (*n* = 2), a further three identifying as Black (*n* = 1), Latino (*n* = 1), or “other” (*n* = 1). All students were participating in return for course credit and the recruitment and data collection procedures were approved by the institution research ethics board.

### 2.2 Materials

Full methodological details have been outlined by Gallagher et al. (2018). Details specifically relevant to the current study are outlined below. Blood pressure and heart rate (HR) were measured using a Finometer Pro hemodynamic monitor (Finapres Medical Systems BV, BT Arnhem, Netherlands).

#### 2.2.1 Life event stress

Life event stress was measured using the 36‐item Life Events for Students Scale (LESS Linden, 1984). This scale was specifically chosen as it is tailored to students in higher education. The scale is comprised of life events that students may have encountered over the past year. Participants are required to indicate (1) the number of life events experienced and (2) their rating of perceived stressfulness of each event on a scale of 1 (Not at all) to 4 (Very). Life event stress was measured as the number of stressful life events experienced.

#### 2.2.2 Stress‐tasks

The personally salient stress‐task required participants to prepare and deliver a speech verbally on three of their worst, and three of their best qualities (Bosch et al., 2009; van Eck et al., 1996), thereby ensuring that this task was highly personally relevant.

The personally non‐salient stress‐task was the paced auditory serial addition task (PASAT Gronwall, 1977). The PASAT, where participants are required to engage in mental arithmetic, returning their answers verbally, offered an equivalent verbal stress‐task, but without the personally salient element. Full details of both tasks are outlined by Gallagher et al. (2018).

#### 2.2.3 Psychological ratings

To measure if the stress‐tasks were perceived as stressful, a number of rating scales were completed in advance and after completion of the stress‐tasks. Participants were asked to rate how difficult, stressful, engaging, and embarrassing they expected to find, and then found, each task on a 7‐point Likert scale, from “not at all” to “very”.

### 2.3 Procedure

Full procedural details are outlined by Gallagher et al. (2018). In summary, following a 20‐minute acclimatization period, cardiovascular measures were recorded for a 10‐minute “vanilla baseline” period (Jennings et al., 1992), followed by completion of the two stress‐tasks, with a 2‐min interval between each task. The order of tasks was counter‐balanced across the procedure.

## 3 RESULTS

### 3.1 Data reduction

Phase‐level means were computed for SBP, DBP, and HR during baseline, PASAT, and speech tasks. Delta scores, between task levels and mean baseline were computed for use in regression analyses reflecting reactivity to the personally non‐salient stress (PASAT), personally salient stress (speech), and to both tasks together (overall reactivity).

### 3.2 Manipulation checks

#### 3.2.1 Psychological ratings

Paired samples *t*‐tests confirmed that participants expected to find the PASAT more difficult and stressful than the speech task (both *p*s < .001), but expected to find the speech task to be more engaging than the PASAT (*p* < .001). There were no differences in how embarrassing participants expected to find both tasks (*p* = .075).

Following the tasks, participants reported that they found the PASAT more difficult and more stressful (both *p*s < .001) than the speech task. In addition they found the speech task to be more embarrassing than the PASAT (*p* = .002), but reported both tasks to be equally engaging (*p* = .068). These findings suggest that the speech task was experienced as more personally salient than the PASAT, given the difference in perceived embarrassment experienced between the two tasks. Table 1 shows the mean (with *SD*) ratings returne pre‐ and post‐task.

**TABLE 1:** Mean (with *SD*) psychological ratings of task expectations and experience

	Speech		PASAT		*p*
	*M*	*SD*	*M*	*SD*	
*Expectations (pre‐task)*					
Difficulty	2.74	1.38	3.83	1.42	<.001
Stressful	2.83	1.63	3.78	1.50	<.001
Engaging	3.46	1.38	2.80	1.64	.075
Embarrassing	3.26	1.70	2.95	1.92	<.001
*Experience (post‐task*)					
Difficulty	4.47	1.35	5.09	1.03	<.001
Stressful	4.19	1.44	4.66	1.21	<.001
Engaging	2.57	1.57	2.88	1.92	.068
Embarrassing	4.35	1.69	3.68	1.82	.005

#### 3.2.2 Cardiovascular reactivity

A series of 2 × 3 mixed ANOVAs were conducted to examine if both tasks were successful in perturbing the cardiovascular system, as well as examining if the order of task presentation moderated the increase from baseline levels. ANOVA confirmed main effects for phase on SBP, *F*(1.82, 256.87) = 168.66, *p* < .001, $\eta_{p}^{2}$ = .55, DBP, *F*(1.77, 249.07) = 170.85, *p* < .001, partial $\eta_{p}^{2}$ = .55, and HR, *F*(2,278.53) = 15.44, *p* < .001, $\eta_{p}^{2}$ = .10. As can be seen in Table 2, and confirmed by pairwise comparisons with Bonferroni adjustment, levels during both tasks were statistically significantly higher than baseline (all *p*s < .001). There were no main effects for task order, confirming that across the study phases, cardiovascular levels were equivalent regardless of whether participants engaged in the PASAT or speech task first (all *p*s > .05).

**TABLE 2:** Mean (with *SD*) blood pressure and heart rate during baseline and both tasks

	Baseline		PASAT		Speech	
	*M*	*SD*	*M*	*SD*	*M*	*SD*
SBP	121.54	9.48	138.75	15.56	142.33	17.05
DBP	73.39	6.93	84.67	9.76	87.09	10.54
HR	78.69	10.66	82.45	11.42	84.67	11.66

There was a phase × task order interaction on SBP, *F*(1.82, 256.87) = 4.20, *p* = .016, $\eta_{p}^{2}$ = .03, and DBP, *F*(1.77, 24,907) = 3.62, $\eta_{p}^{2}$ = .03, but not on HR, *F*(2, 280) = .20, *p* = .82. Participants showed lower cardiovascular elevation to the PASAT when it was presented second, but absolute levels during the speech task were equivalent regardless of whether speech was presented first or second. Two 2 × 2 mixed ANOVAS were conducted to confirm the effect of task order in response to the tasks. Delta scores were computed for PASAT and speech, by subtracting baseline levels from task levels, returning a two‐level within‐subjects factor of task reactivity; reactivity to PASAT, reactivity to speech. The between‐subjects factor was task order. ANOVA revealed a significant reactivity × task order interaction for SBP, *F*(1,141) = 6.48, *p* = .012, $\eta_{p}^{2}$ = .04, and DBP, *F*(1, 141) = 5.79, *p* = .017, $\eta_{p}^{2}$ = .04. Analyses revealed that the difference between reactivity to the PASAT and speech was only influenced by doing the PASAT first; when speech was first, reactivity to the PASAT and speech were equivalent. When PASAT was completed first, reactivity to the speech was higher than when speech was first. Consequently, task order was included as a control variable in regression models examining SBP and DBP.

### 3.3 Life event stress and CVR to the combined stressors

As much of the reactivity literature is based on computing reactivity across a number of tasks, or to a variety of tasks, a series of linear regression models were conducted to examine if life event stress was associated with reactivity averaged across both tasks. This partially repeats the analyses as reported by Gallagher et al. (2018). Age, sex, task order (not entered in the model examining HR), and the relevant baseline value was entered in the first step, LESS total events was entered in the second step. While the overall regression models was not significant for SBP, *F*(5, 126) = 1.79, *p* = .120, life event stress was negatively associated with SBP reactivity, *β* = −.21, *p* = .016, with higher frequency of life event stress associated with lower SBP reactivity.

For DBP, the overall model with all predictors was significant, *F* (5, 126) = 3.73, *p* = .003. As with SBP, life event stress was negatively associated with DBP reactivity, *β* = −.28, *p* = .001.

For HR, while the overall model was significant, *F*(4, 129) = 1318.55, *p* < .001, HR, life event stress was not significantly associated with HR reactivity, with only baseline HR adding significantly to the model predicting HR reactivity, *β* = −.58, *p* < .001. Therefore, while life event stress was associated with blunted blood pressure responding, there was no association with HR reactivity.

### 3.4 Life event stress and CVR to both personally salient and personally non‐salient stressors

To establish if it is the case that blunted reactivity only arises where stressors have no personal relevance, separate regression models were conducted with reactivity to both tasks as separate outcome variables. The models are a replication of those presented above, with only the outcome variable changed. Separate regression models are presented for reactivity to personally non‐salient stress (reactivity to PASAT) and personally salient stressors (reactivity to speech).

The regression models with reactivity to PASAT as outcome variable identified that life event stress was not associated with SBP reactivity to PASAT, *β* = −.15, *p* = .102, but was negatively associated with DBP reactivity, *β* = −.20, *p* = .023, although the overall model with DBP reactivity to speech as outcome was not significant, *F*(5, 126) = 1.57, *p* = .174. As with the regression model on overall HR reactivity, while the model with all predictors was significant, *F*(4, 127) = 11.45, *p* < .001, it was only baseline HR that significantly contributed to the model, *β* = −.46, *p* < .001.

For the personally salient stress, findings were more conclusive. Life event stress was negatively associated with both SBP reactivity to speech, *F*(5, 126) = 2.78, *p* = .02, adjusted *R* ^2^ = .06, *β* = −.20, *p* = .021, and DBP reactivity to speech, *F*(5, 126) = 5.51, *p* < .001, adjusted *R* ^2^ = .15, *β* = −.24, *p* = .004. As with the earlier models examining overall HR reactivity and HR reactivity to personally non‐salient stress, while the overall model was significant, *F*(4, 129) = 13.41, *p* < .001, it was only baseline HR that significantly contributed to the model, *β* = −.54, *p* < .001. Table 3 shows a summary of the regression models.

**TABLE 3:** Summary of regression models for systolic blood pressure, diastolic blood pressure, and heart rate

		Systolic blood pressure			Diastolic blood pressure			Heart rate		
		B	SE B	*β*	B	SE B	*β*	B	SE B	*β*
**Overall**										
Block 1	(Constant)	34.39	17.54		35.52	9.58		46.03	10.41	
	Age	−.45	.46	−.09	−.58	.28	−.18*	.37	.36	.07
	Sex	−1.17	2.05	−.05	−1.47	1.26	−.10	−.04	1.68	.00
	Task Order	−2.57	2.00	−.11	−.84	1.23	−.06	–	–	–
	Baseline	.00	.11	.00	−.10	.10	−.09	−.62	.08	−.58***
Block 2	(Constant)	44.33	17.68		42.94	9.51		46.54	10.52	
	Age	−.53	.45	−.10	−.64	.27	−.20*	.36	.36	.07
	Sex	−1.25	2.01	−.05	−1.52	1.21	−.10	−.07	1.69	.00
	Task Order	−2.71	1.96	−.12	−.97	1.18	−.07	–	–	–
	Baseline	−.02	.11	−.02	−.12	.10	−.11	−.62	.08	−.58***
	LESS	−.65	.26	−.21*	−.52	.16	−.27**	−.09	.22	−.03
**PASAT**										
Block 1	(Constant)	1.60	18.67		7.18	10.60		32.90	11.96	
	Age	−.14	.49	−.03	−.20	.31	−.06	.66	.41	.12
	Sex	−.85	2.19	−.03	.21	1.39	.01	−1.26	1.95	−.05
	Task Order	1.28	2.13	.05	1.76	1.36	.11			
	Baseline	.15	.12	.11	.07	.11	.06	−.52	.09	−.46***
Block 2	(Constant)	8.79	19.06		13.06	10.73		33.37	12.09	
	Age	−.20	.49	−.04	−.25	.31	−.07	.65	.41	.12
	Sex	−.91	2.17	−.04	.17	1.37	.01	−1.30	1.96	−.05
	Task Order	1.18	2.11	.05	1.65	1.34	.11	–	–	–
	Baseline	.13	.12	.10	.05	.11	.04	−.52	.09	−.45***
	LESS	−.47	.28	−.14	−.41	.18	−.20*	−.08	.25	−.02
**Speech**										
Block 1	(Constant)	67.18	23.35		63.853	12.923		56.86	12.95	
	Age	−.76	.61	−.11	−.965	.379	−.213*	.10	.45	.02
	Sex	−1.50	2.73	−.05	−3.156	1.692	−.155	.26	2.09	.01
	Task Order	−6.43	2.66	−.21*	−3.439	1.656	−.174*	–	–	–
	Baseline	−.15	.15	−.09	−.267	.133	−.168*	−.68	.10	−.54***
Block 2	(Constant)	79.86	23.58		72.817	12.927		57.41	13.08	
	Age	−.87	.60	−.12	−1.039	.369	−.229*	.09	.45	.02
	Sex	−1.60	2.69	−.05	−3.213	1.645	−.158	.22	2.10	.01
	Task Order	−6.60	2.61	−.21*	−3.597	1.610	−.182*	–	–	–
	Baseline	−.18	.15	−.10	−.296	.130	−.186*	−.68	.10	−.54***
	LESS	−.83	.35	−.20*	−.630	.216	−.237**	−.09	.27	−.03

*Note*: Age, sex, and relevant baseline were entered as covariates for each module. Task order was included as covariate for models examining systolic blood pressure and diastolic blood pressure.

*p < .05, ** *p* < .01, *** *p* < .001

To test if blunted reactivity may reflect a stable pattern of individual difference, the association between reactivity to personally salient and personally non‐salient stress was examined. Pearson's *r* indicated that there was a high positive correlation between SBP reactivity to personally salient and non‐salient stress, *r*_p_ = +.33, *p* < .001, as well as DBP reactivity to personally salient and non‐salient stress, *r*_p_ = +.28, *p* < .001, and HR reactivity to personally salient and non‐salient stress, *r*_p_ = +.50, *p* < .001.

## 4 DISCUSSION

The present study identified that blunted cardiovascular reactions to stress are associated with life event stress in a sample of young, healthy adults, regardless of the personal salience of the laboratory task used. The findings contradict Bourassa and Sbarra's (2022) suggestion that the type of task chosen to assess reactivity in the laboratory may be an explanatory factor in the now large literature identifying blunted cardiovascular reactions to stress as associated with ill‐health. This addressed a limitation in Bourassa and Sbarra's study where the findings were based on a laboratory task that was not experienced as stressful for the entire sample; only a subset of their sample, those who reported experiencing high divorce‐related stress, showed elevated cardiovascular measures during the divorce recall task compared to baseline measures. Using two tasks that required verbal return of responses, the present study employed the PASAT and a self‐relevant speech task to test if only tasks with low personal salience would be associated with blunted reactions. It identified a high correlation between reactivity to both the personally salient and non‐salient tasks. In addition, those reporting high life event stress showed blunted reactions to the combined task (as reported by Gallagher et al., 2018 and confirmed in the present study), as well as showing blunted reactions to both the personally salient and non‐salient tasks.

A large body of literature now exists showing that blunted cardiovascular reactions to stress are associated with a range of negative health and psychological outcomes (e.g., O'Riordan et al., in press; Phillips et al., 2013; Whittaker et al., 2021). However, as correctly identified by Bourassa and Sbarra (2022), much of the research employed laboratory tasks with low personal emotional relevance, such as mental arithmetic and Stroop. These are purposely designed to have low personally emotional salience but are still perceived as highly stressful, and regularly contain socially evaluative conditions, such as overt marking by the experimenter. However, a limitation of the Bourassa and Sbarra study was that the “stressor” task chosen was not stressful for the whole sample. That is, across the group, the divorce recall task did not elevate cardiovascular parameters and so was not successful in eliciting a cardiovascular response to the psychological stressor. This is usually a fundamental assumption of tasks used as stressors in reactivity research. It is difficult to argue that blunted reactions to stress arise due a lack of personal emotional salience when the task chosen was not perceived as stressful for the sample. Usually, the stressor experienced elicits a cardiovascular response for the whole sample and it is only a subset of the sample that show no or low reactivity. Our findings identify that blunted reactivity to stress is present to both personally salient and non‐salient stress, using a task that elicited a cardiovascular response for the whole sample.

Our study addressed an empirical question relating to blunted reactivity to stress; are blunted reactions to stress only evident on personally non‐salient stress tasks? Using life event stress, a psychological construct that has consistently shown negative associations to laboratory stressors (e.g., Carroll et al., 2005; Musante et al., 2000; Phillips, Burns, et al., 2005; Tyra et al., 2020), we showed that blunted reactivity was present to both personally salient and non‐salient stress, with negative associations between reactivity and life event stress. This conclusion suggests that researchers should exercise caution when interpreting patterns of exaggerated and blunted reactivity where the stress tasks used have not elicited a stress response overall, and that this manipulation check should be a fundamental initial component of stress reactivity analyses.

However, as always, the present study is not without some limitations. As the data were reanalyzed from a previous study, we did not include specific measure of the personal salience of the task. However, the tasks used were such that one task was, by nature, personally salient while the other was not. Further, post‐task ratings of embarrassment showed that participants were more embarrassed during the speech task compared to the PASAT; but were equally engaged during both tasks. This highlights the construct validity of the tasks used, which is important given that this paper is based on secondary data analyses. Further, both tasks have been used previously, with the personally salient stressor shown to contain high social evaluative threat (Bosch et al., 2009; van Eck et al., 1996).

It must also be acknowledged that in both the present study and in Bourassa and Sbarra's (2022) study, personal salience was not the only distinguishing difference between the two tasks. Both studies used a numerical task (PASAT and mental arithmetic) and a speech task (speaking about personal qualities and divorce recall). Essentially, these tasks could be considered as arithmetic (PASAT) versus verbal (speech) in both studies, with neither study offering a clear experimental contr. Consequently, the findings reported need to be replicated using a stressor with equivalent tasks demands, physically and cognitively, with just the personal salience of the tasks altered between participants (i.e., two speech tasks with varying personal salience in the topics).

The present study did not reveal a consistent pattern of blunted reactivity across all cardiovascular parameters; however, this is common in reactivity research and may reflect characteristics of the sample studied, for example age, hypertension status et cetera. However, the sample in the present study were specifically chosen to ensure young adults were examined, with no self‐reported existing cardiovascular disease including hypertension status. Future research needs to identify if different outcomes arise from blunted reactivity on different cardiovascular parameters. A recent systematic review and meta‐analysis (O'Riordan et al., in press) confirmed that blunted HR reactivity is the most common reactivity predictor of negative outcomes. It may be the case that blunted cardiovascular reactions to stress signal different outcomes depending on whether they relate to HR specifically, or blood pressure.

While the personally salient task did not relate directly to the construct being measured (i.e., life events stress), it does offer a rebuttal to Bourassa and Sbarra's (2022) argument that it is important to choose personally salient tasks in reactivity research, particularly within the blunted model of reactivity. In this study, the stress‐task was perceived as stressful for the entire sample, with the personally salient task perceived as more embarrassing. Future research may address the limitations in both studies and employ a more personally salient task that relates directly to the construct being measured, but one that is also stressful for the entire sample. However, to conclude, this re‐analysis of data queries whether the stress‐task's personal salience used in reactivity studies is, in fact, an important factor to consider, with our findings showing that participants show similar levels of cardiovascular responding to tasks of both high and low personal salience.

## AUTHOR CONTRIBUTIONS

**Siobhán Howard:** Conceptualization; formal analysis; investigation; writing – original draft. **Stephen Gallagher:** Data curation; investigation; methodology; writing – review and editing. **Annie T. Ginty:** Conceptualization; investigation; writing – review and editing. **Anna C. Whittaker:** Conceptualization; investigation; writing – review and editing.

## Data Availability

Data available on request from the authors.
